# Association between the cut-off value of the first trimester fasting plasma glucose level and gestational diabetes mellitus: a retrospective study from southern China

**DOI:** 10.1186/s12884-022-04874-x

**Published:** 2022-07-04

**Authors:** Jia-Ning Tong, Yi-Xuan Chen, Xiao-Nian Guan, Kan Liu, Ai-Qi Yin, Hua-Fan Zhang, Lin-Lin Wu, Jian-Min Niu

**Affiliations:** Department of Obstetrics, The First School of Clinical Medicine, Shenzhen Maternity & Child Healthcare HospitalSouthern Medical UniversityFutian District, Hongli Road, Shenzhen, Guangdong Province China

**Keywords:** First-trimester FPG, Cut-off values, GDM, Metabolic diseases in pregnancy, Adverse pregnancy outcomes

## Abstract

**Purpose:**

Our previous studies have suggested that the first trimester fasting plasma glucose (FPG) level is associated with gestational diabetes mellitus (GDM) and is a predictor of GDM. The aim of the present study was to provide valuable insights into the accuracy of the first trimester FPG level in the screening and diagnosis of GDM in southern China.

**Methods:**

This retrospective study included pregnant women who had their first trimester FPG level recorded at 9–13^+6^ weeks and underwent screening for GDM using the 2-h 75 g oral glucose tolerance test (OGTT) between the 24th and 28th gestational weeks. Differences between the GDM and non-GDM groups were assessed by Student’s t test and the chi-squared test according to the nature of the variables. A restricted cubic spine was used to explore the relationship between the first trimester FPG level and the odds ratio (OR) of GDM in pregnant women. Cut-off values of first trimester FPG were determined using receiver operating characteristic (ROC) curves and the area under the curve (AUC), and 95% confidence intervals (CIs), the positive predictive value (PPV) and the negative predictive value (NPV) were calculated.

**Results:**

The medical records of 28,030 pregnant women were analysed, and 4,669 (16.66%) of them were diagnosed with GDM. The average first trimester FPG level was 4.62 ± 0.37 mmol/L. The OR of GDM increased with increasing first trimester FPG levels and with a value of first trimester FPG of approximately 4.6 mmol/L, which was equal to 1 (Chi-Square = 665.79, P < 0.001), and then started to increase rapidly afterwards. The ROC curve for fasting plasma glucose in the first trimester (4.735 mmol/L) for predicting gestational diabetes mellitus in pregnant women was 0.608 (95% CI: 0.598–0.617), with a sensitivity of 0.490 and a specificity of 0.676.

**Conclusion:**

Based on the research, we recommend that all pregnant women undergo FPG testing in the first trimester, particularly at the first antenatal visit. Furthermore, we suggest that the risks of GDM should be given increased attention and management as soon as the first trimester FPG value is more than 4.7 mmol/L. First trimester FPG levels should be considered a screening marker when diagnosing GDM in pregnant women but this needs to be confirmed by more prospective studies. These factors may have a significant impact on the clinical treatment of pregnant women.

## Introduction

Gestational diabetes mellitus (GDM) is one of the most common medical diseases related to pregnancy. It was previously defined as “hyperglycaemia first detected during pregnancy” [1]. According to the WHO, approximately 16% of pregnant women worldwide are affected by GDM [2]. GDM usually manifests in the second half of pregnancy and is caused by extreme physiological insulin resistance. Early diagnosis and treatment of GDM is extremely important because GDM can lead to several severe maternal-foetal complications, such as neonatal hypoglycaemia, birth injuries, macrosomia, shoulder dystocia, respiratory distress syndrome, childhood obesity and perinatal mortality [3]. Despite the worldwide prevalence and severity of GDM, a universally accepted screening test is lacking. Screening tests and diagnostic criteria vary widely among clinicians and across geographic areas [1]. Thus, the optimal method to screen for GDM in the first trimester remains unclear.

The ACOG (The American College of Obstetricians and Gynecologists)and ADA(American Diabetes Association) recommend that all pregnant women, regardless of their risk factors, should be screened for GDM by an OGTT(Oral Glucose Tolerance Test) at 24 to 28 weeks of gestation [4, 5]. In 2010, the IADPSG (International Association of Diabetes and Pregnancy Study Groups) also recommended a 75-g OGTT test at 24–28 weeks of gestation for the diagnosis of GDM in all pregnant women with no apparent history of diabetes [6]. However, some studies have pointed out that OGTTs cannot be widely implemented because of the complexity of the tests, the need for a prior appointment, the long waiting time, and the low cost-effectiveness [7]. Doctors are thus attempting to find a more acceptable alternative strategy for the diagnosis of GDM to reduce the number of pregnant women who need to undergo an OGTT.

In this regard, FPG (Fasting Plasma Glucose) has been reported to have good efficacy as a screening test for GDM, especially at low thresholds, which has a strong influence on the exclusion of GDM in women. High-precision FPG tests can reduce the burden on the laboratory and save resources because it may be very difficult to carry out 75-g OGTTs with a large population and limited resources [8]. Previous studies have shown that FPG can be used to predict the risk of GDM in the third trimester, but there are significant differences among geographical regions of the world [7, 8]. Compared with the use of OGTTs, the use of FPG is easy to manage, well tolerated, reliable and has good repeatability; FPG also changes minimally throughout the entire pregnancy [9]. However, the usefulness of FPG in predicting GDM is not widely recognized because of the different diagnostic criteria, the choice of gestational age and differences related to race. There are no recognized diagnostic criteria for FPG in pregnant women [9, 10]. The use of first trimester FPG for screening GDM lacks related research with large samples in southern China, where the prevalence of GDM is different from that in northern China because of cooking habits, flavour styles and so on [11, 12]. Southerners like sweets and eat rice, whereas northerners prefer salty food. Therefore, it is necessary to establish some evidence for the use of first trimester FPG and to delineate its optimal cut-off value for diagnosing GDM in southern China. In our study, we attempted to use the IADPSG standard [6] to assess the sensitivity and specificity of FPG in the diagnosis of GDM to avoid the implementation of many OGTT tests in southern China.

## Methods

### Subjects

This retrospective study included pregnant women who delivered between 1 June 2017 and 31 December 2019 at Shenzhen Maternal and Childcare Hospital in Shenzhen, southern China. Pregnant women with first trimester FPG levels > 7 mmol/L were excluded. The purpose of FPG evaluation in the centre was to exclude women with pregestational diabetes. Then, we excluded pregnant women who were younger than 18 years old, had multiple pregnancies, and had pregnancies conceived by assisted reproductive technology. Those whose first trimester FPG values (FPG value between 9–13^+6^ weeks of gestation) were available with complete data for outcomes included 48,444 persons, which was also in accordance with our last study^17^. Of the 48,444 persons, only pregnant women with available OGTT results at 24–28 gestational weeks with complete data for outcomes were included.

**Fig. 1:**
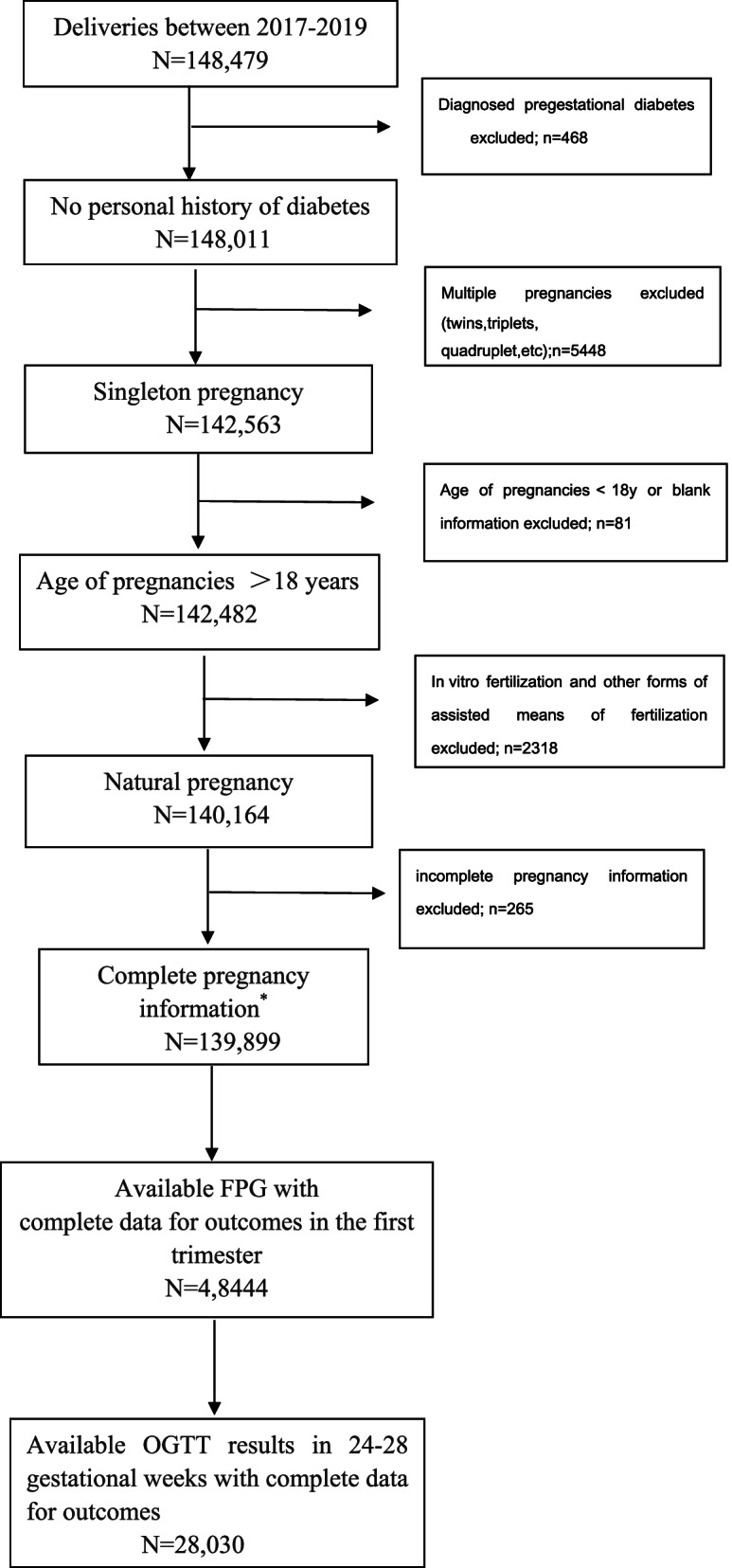
Flow chart of the selection process of the survey

Ultimately, 28,030 pregnant women were included in our study (Fig. 1). No treatment intervention (even lifestyle changes) was conducted in this cohort, and the results were reliable. The study was reviewed and approved by the Ethical Review Boards of Shenzhen Maternal and Childcare Hospital (Approval number: Shenzhen Maternal and Child Ethics Review No. 23; Approval date: 2017–04-07).

### Data collection and definitions

Demographic information and medical data, such as maternal age, pregestational body mass index (BMI), first trimester FPG value (samples collected before 10 am every day), and 0-, 1- or 2-h plasma glucose values using a 2-h 75 g OGTT between 24–28 weeks gestation, were collected. The first trimester FPG and OGTT results were measured using the enzyme electrode method (DXC800, Beckman). First-trimester FPG was measured using venous blood samples after at least 8 h at the first visit and divided into seven groups according to the HAPO study (< 4.2 mmol/L, 4.2–4.4 mmol/L, 4.5–4.7 mmol/L, 4.8–4.9 mmol/L, 5.0–5.2 mmol/L, 5.3–5.5 mmol/L, ≥ 5.6 mmol/L). GDM was defined as FPG, 1- or 2-h plasma glucose(PG) values of ≥ 5.1, ≥ 10.0, or ≥ 8.5 mmol/L, respectively, using 2-h 75 g OGTT values between 24–28 weeks gestation by the IADPSG standard [6]. The study population was stratified according to GDM status by the IADPSG standard: GDM and non-GDM.

### Statistical methods

Data are presented as the mean ± standard deviation (SD) for continuous variables and numbers (percentages) for categorical variables. Differences between the GDM and non-GDM groups were assessed by Student’s t test and the chi-squared test according to the nature of the variables. A restricted cubic spine was used to explore the relationship between first trimester FPG values and the odds ratio (OR) of GDM in pregnant women. Cut-off values of first trimester FPG were determined using receiver operating characteristic (ROC) curves and the area under the curve (AUC), and 95% confidence intervals (CIs), the positive predictive value (PPV) and the negative predictive value (NPV) were calculated. All statistical analyses were carried out using R software (version 4.0.3, https://www.r-project.org/). A two-tailed *P* value < 0.05 was considered statistically significant.

## Results

### Clinical characteristics of pregnant women between the GDM and non-GDM groups

Table 1 displays the different characteristics of the pregnant women between the GDM and non-GDM groups. Among 28,030 pregnant women, 4,669 (16.66%) were diagnosed with GDM, which was similar to that reported by the WHO [2]. The maternal age was 31.01 ± 4.12 years, the gestational BMI was 20.73 ± 2.41 kg/m^2^, and both the maternal age and pregestational BMI in women with GDM were significantly higher than those in non-GDM women (*P* < 0.001). Among the GDM group, women aged 30–34 years accounted for the largest number (42.13%), while women aged less than 30 years accounted for the largest number (42.87%) in the non-GDM group. The first trimester FPG level was 4.62 ± 0.37 mmol/L. Interestingly, the results showed that the proportion of GDM increased as the first trimester FPG level increased when it was lower than 4.7 mmol/L (*P* < 0.001). When the first trimester FPG value was between 4.2–4.4 mmol/L, the proportion of GDM (6.47% for a first trimester FPG value of < 4.2 mmol/L to 17.95% for a first trimester FPG value of 4.2 mmol/L- 4.4 mmol/L) sharply increased until the first trimester FPG value reached 4.5–4.7 mmol/L (25.68%, all P < 0.001), and the proportion of GDM peaked. This trend is consistent with the HAPO study, in which an FPG value ≤ 4.4 mmol/L (80 mg/dL) was associated with a lower risk of some adverse outcomes to some degree [6]. The proportion of GDM decreased in the groups with first trimester FPG levels of 4.8–4.9 mmol/l, 5.0–5.2 mmol/L, 5.3–5.5 mmol/L and ≥ 5.6 mmol/L, which were 23.82%, 15.40%, 6.83% and 3.86%, respectively (*P* < 0.001).

**Table 1:** Clinical characteristics in pregnant women between GDM and Non-GDM

**Features**	**Overall(*N* = 28,030)**	**N**on**-GDM(*N* = 23,361)**	**GDM(*N* = 4669)**	**P**
Maternal age, years, mean ± SD	31.01 ± 4.12	30.71 ± 4.03	32.51 ± 4.25	< 0.001
< 30, years, n (%)	11,235 (40.08)	10,016 (42.87)	1219 (26.11)	< 0.001
30–34, years, n (%)	11,155 (39.80)	9188 (39.33)	1967 (42.13)	
35–39, years, n (%)	4752 (16.95)	3567 (15.27)	1185 (25.38)	
≥ 40, years, n (%)	888 (3.17)	590 (2.53)	298 (6.38)	
Pregestational BMI, kg/m^2^, mean ± SD	20.73 ± 2.41	20.62 ± 2.37	21.31 ± 2.52	< 0.001
< 24 kg/m^2^, n (%)	25,485 (90.92)	21,437 (91.76)	4048 (86.70)	< 0.001
≥ 24 kg/m^2^, n (%)	2545 (9.08)	1924 (8.24)	621 (13.30)	
First trimester FPG, mmol/L, mean ± SD	4.62 ± 0.37	4.59 ± 0.36	4.75 ± 0.43	< 0.001
< 4.2 mmol/L,n(%)	2812 (10.03)	2510 (10.74)	302 (6.47)	< 0.001
4.2–4.4 mmol/L, n (%)	6306 (22.50)	5468 (23.41)	838 (17.95)	
4.5–4.7 mmol/L, n (%)	8802 (31.40)	7603 (32.55)	1199 (25.68)	
4.8–4.9 mmol/L, n (%)	6110 (21.80)	4998 (21.39)	1112 (23.82)	
5.0–5.2 mmol/L, n (%)	2773 (9.89)	2054 (8.79)	719 (15.40)	
5.3–5.5 mmol/L, n (%)	859 (3.06)	540 (2.31)	319 (6.83)	
≥ 5.6 mmol/L,n(%)	368 (1.31)	188 (0.80)	180 (3.86)	
OGTT at 24 ~ 28 gestational weeks				
0 h OGTT, mmol/L, mean ± SD	4.35 ± 0.39	4.29 ± 0.32	4.64 ± 0.54	< 0.001
1 h OGTT, mmol/L, mean ± SD	7.446 ± 1.59	7.11 ± 1.31	9.20 ± 1.73	< 0.001
2 h OGTT, mmol/L, mean ± SD	6.62 ± 1.30	6.32 ± 1.00	8.12 ± 1.55	< 0.001

^*^The category of the first-trimester FPG was according to the HAPO study

### The relationship between first trimester FPG values and the odds ratio (OR) of GDM in pregnant women

**Fig. 2:**
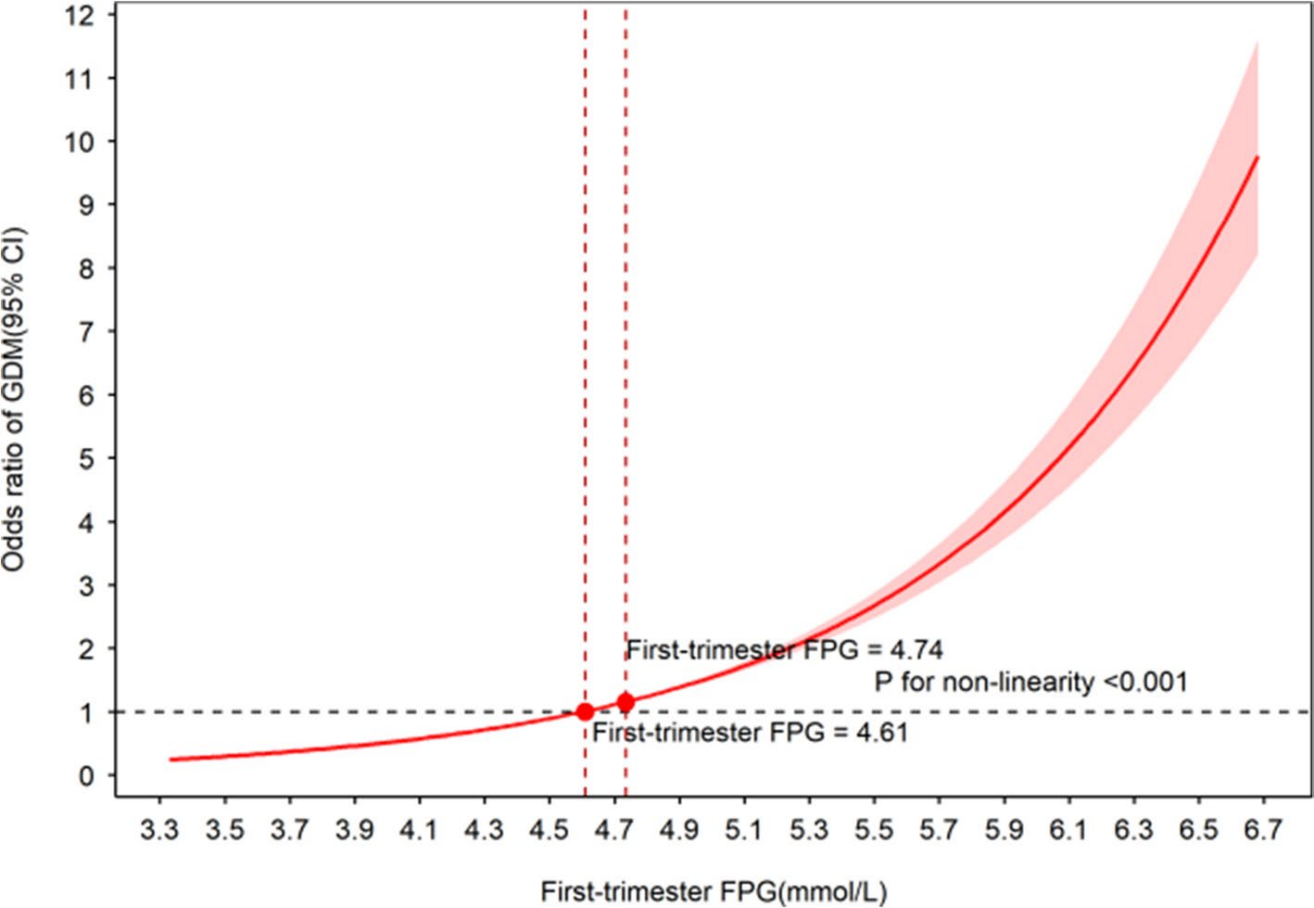
The association between the odds ratio (OR) of GDM and levels of first trimester FPG (mmol/L)

**Fig. 3:**
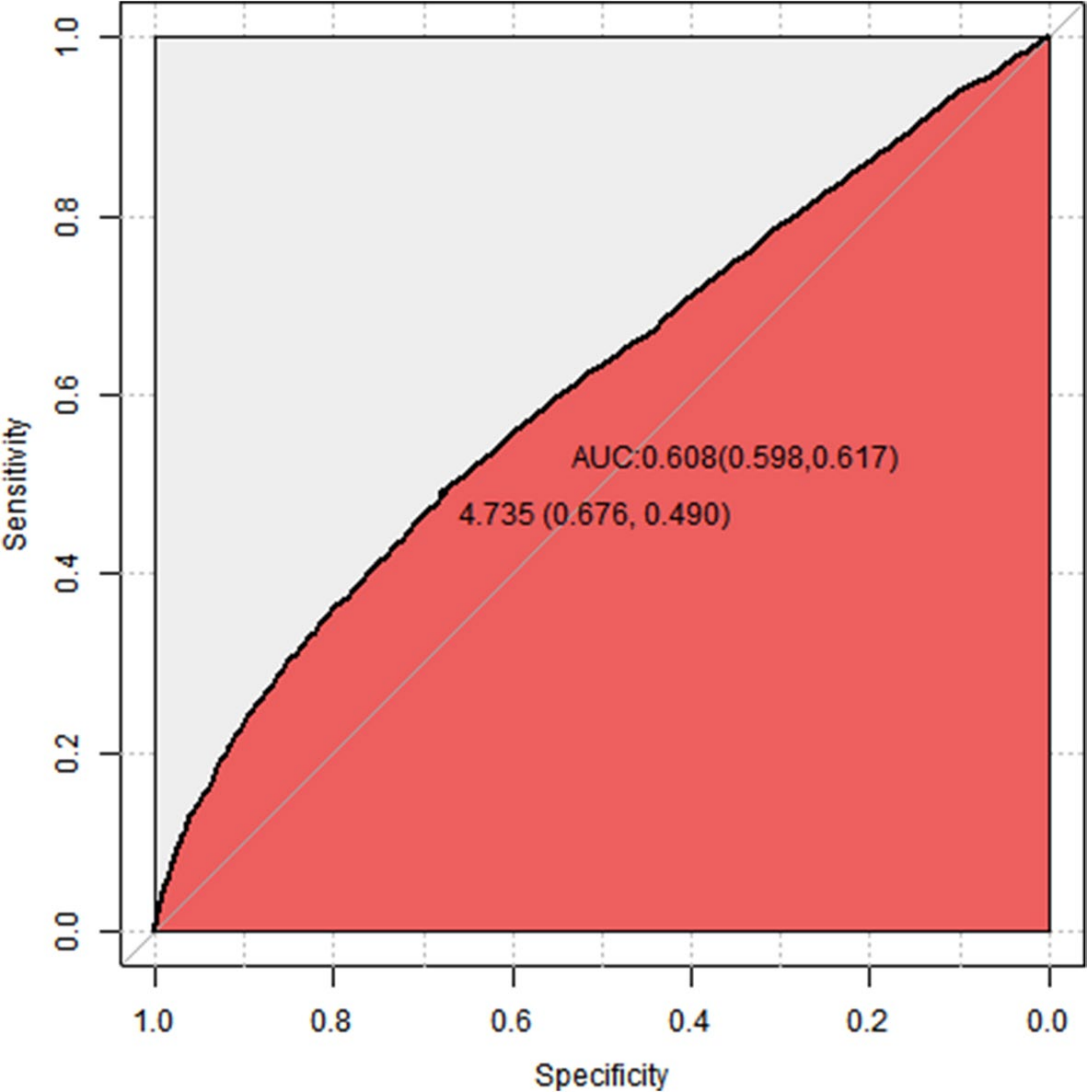
ROC curves for fasting plasma glucose levels at the first trimester in predicting gestational diabetes mellitus in pregnant woman

In Fig. 2, we used a restricted cubic spine to explore the relationship between first trimester FPG values and the odds ratio (OR) of GDM. The OR of identifying GDM increased with increasing first trimester FPG values and with a first trimester FPG value of approximately 4.6 mmol/L, which was equal to 1 (Chi-Square = 665.79, *P* < 0.001), and then started to increase rapidly afterwards. Different fasting plasma glucose levels in the first trimester as a predictor for gestational diabetes mellitus are shown in Table 2. Figure 3 shows the ROC curves for fasting plasma glucose in the first trimester for predicting gestational diabetes mellitus in pregnant women, and the AUC was 0.608 (95% CI: 0.598–0.617) with a sensitivity of 0.490 and a specificity of 0.676, which was similar to some studies [10, 13].

**Table 2:** Different Fasting plasma glucose at the first trimester as a predictor for gestational diabetes mellitus

Cut off value	Accuracy	Sensitivity	Specificity	PPV	NPV
4.6	0.534	0.624	0.516	0.205	0.873
4.7	0.616	0.527	0.633	0.223	0.870
4.8	0.682	0.431	0.733	0.244	0.866
4.9	0.736	0.344	0.814	0.270	0.861
5.0	0.778	0.261	0.881	0.305	0.856
5.1	0.803	0.194	0.924	0.339	0.852
5.2	0.818	0.141	0.954	0.378	0.847
5.3	0.826	0.100	0.972	0.413	0.844
5.4	0.830	0.068	0.982	0.434	0.841
5.5	0.833	0.048	0.989	0.475	0.839
5.6	0.833	0.032	0.993	0.482	0.837
5.7	0.834	0.024	0.996	0.531	0.836
5.8	0.834	0.016	0.997	0.529	0.835
5.9	0.834	0.011	0.998	0.515	0.835
6.0	0.834	0.008	0.999	0.507	0.834
6.1	0.834	0.007	0.999	0.525	0.834
6.2	0.833	0.005	0.999	0.500	0.834
6.3	0.833	0.004	0.999	0.487	0.834
6.4	0.833	0.002	0.999	0.393	0.834
6.5	0.833	0.002	0.999	0.350	0.834
6.6	0.833	0.002	1.000	0.389	0.834
6.7	0.833	0.000	1.000	0.200	0.833
6.8	0.833	0.000	1.000	0.250	0.834
6.9	0.833	0.000	1.000	0.200	0.833

*PPV* positive predictive value, negative predictive value

### The incidence of GDM by screening and diagnostic criteria of the IADPSG

**Fig. 4:**
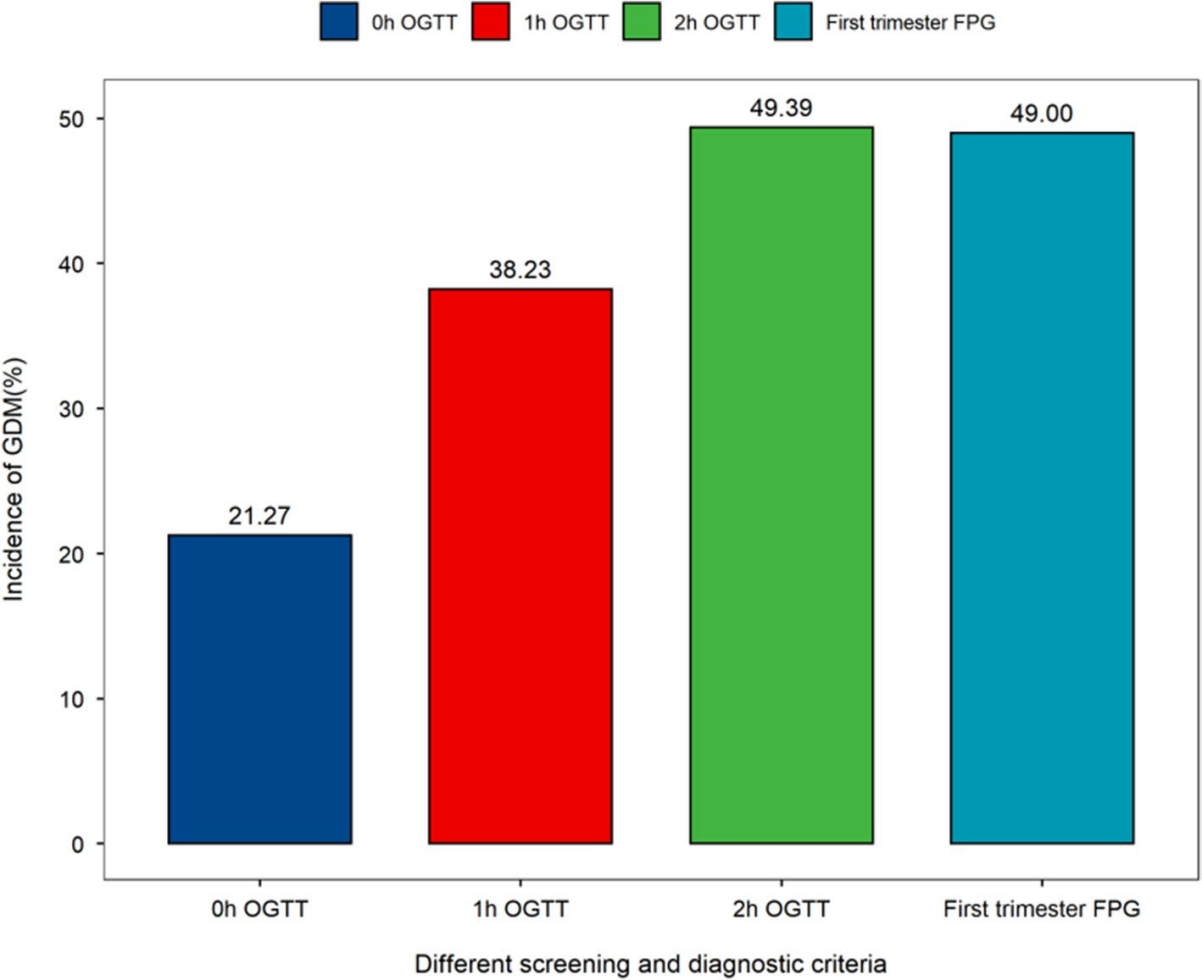
Incidence of GDM by screening and diagnostic criteria by IASPSG. The X-axis showed different screening and diagnostic criteria-axis was rate of GDM by the x-axis screening and diagnostic criteria

First-trimester FPG values greater than or equal to 4.735 mmol/L (first trimester FPG value ≥ 4.735 mmol/L) was set as a screening criterion. Table 3 shows the incidence of GDM by the screening and diagnostic criteria of the IADPSG. According to the IADPSG diagnosis standard of GDM, the incidence of GDM with FPG, 1-h PG and 2-h PG of OGTTs in the overall population was 3.54%, 6.37% and 8.23%, respectively, and Fig. 4 shows the incidence of GDM by the IADPSG criterion. It was indicated that 49.00% of the pregnant women could be diagnosed as having GDM with first trimester FPG values, which overlapped with 21.27%, 38.23% and 49.39% in FPG, 1-h PG and 2-h PG results (OGTT tests) at 24–28 gestational weeks, respectively. However, at the same time, we should consider that it might also have the possibility of being an incorrect diagnosis.

**Table 3:** Incidences of GDM by screening and diagnostic criteria of IADPSG

Screening and Diagnostic criteria	Overall (*N* = 28,030)	Non-GDM (*N* = 23,361)	GDM (*N* = 4669)
OGTT at 24–28 gestational weeks			
0 h OGTT, mmol/L			
FPG < 5.1, mmol/L, n (%)	27,037 (96.46)	23,361(100.00)	3676(78.73)
FPG > = 5.1, mmol/L, n (%)	993 (3.54)	0(0.00)	993(21.27)
1 h OGTT, mmol/L			
1 h OGTT < 10, mmol/L, n (%)	26,245 (93.63)	23,361(100.00)	2884(61.77)
1 h OGTT > = 10.0, mmol/L, n (%)	1785 (6.37)	0(0.00)	1785(38.23)
2 h OGTT, mmol/L			
2 h OGTT < 8.5, mmol/L, n (%)	25,724 (91.77)	23,361(100.00)	2363(50.61)
2 h OGTT > = 8.5, mmol/L, n (%)	2306 (8.23)	0(0.00)	2306(49.39)
First trimester FPG, mmol/L			
First trimester FPG < 4.735, mmol/L, n (%)	18,174(64.84)	15,793(67.60)	2381(51.00)
First trimester FPG > = 4.735, mmol/L, n (%)	9856(35.16)	7568(32.40)	2288(49.00)

## Discussion

In 2008, the HAPO study was conducted with 25,505 pregnant women who underwent 75 g OGTTs at 24 to 32 gestational weeks in 15 centres from nine countries and showed that an elevated FPG value that was lower than the diagnosis level of diabetes in pregnant women was associated with adverse pregnancy outcomes, including GDM, LGA and/or macrosomia, as well as caesarean delivery, and was strongly and consistently associated with birth weight gain and elevated C-peptide levels in cord blood [13]. Another HAPO study further noted that higher FPG levels in the first trimester, which are currently considered to be nondiabetic, increased the risk of adverse pregnancy outcomes [14].

Early diagnosis of GDM is essential to prevent related pregnancy complications. A study in 2009 reported that higher first trimester FPG levels increased the risk of some complications and implied that women with high risks would not receive appropriate attention if they were not diagnosed during the first trimester [14]. Mills et al. found a physiological decrease in FPG levels during normal pregnancy, which indicated that the standard of FPG in pregnant individuals should be different from that in nonpregnant individuals [15]. Our last study also revealed a strong relationship between adverse pregnancy outcomes and GDM. The research reported that the first trimester FPG level was strongly associated with risks of outcomes, including GDM, caesarean section, macrosomia, GHD, primary caesarean section and LGA (all ORs > 1, all Ps < 0.05). Furthermore, the risks of GDM, primary caesarean section and LGA increased with a first trimester FPG level of 4.19–4.63 mmol/L. After adjustments for multiple factors, every increase in the first trimester FPG level was associated with the risk of GDM. With increasing first trimester FPG levels, the risks of GDM increased [16].

The risks that the age of onset of undiagnosed type 2 diabetes in young women during pregnancy decline. The importance of screening for and properly managing GDM cannot be underestimated because GDM can cause severe maternal and infant complications [1, 11]. However, it is estimated that in some countries with limited health care resources, the lack of universal screening may miss up to 43% of GDM patients [1]. Although the OGTT is the gold standard diagnostic test for GDM, it is associated with several potential limitations, such as high costs and laboratory requirements [8]. Therefore, screening all pregnant women with OGTTs can be difficult. Although FPG levels are not the gold standard for diagnosing GDM, measuring first trimester FPG may be critical for screening for individuals with previously undiagnosed diabetes. FPG tests have been proposed as a screening test for GDM because they are less time consuming and more user friendly and reduce the medical costs associated with universal oral glucose tolerance testing [8, 14, 17]. Therefore, it is important to determine the diagnostic performance and optimal cut-off value of FPG for GDM screening, especially for pregnant women in their first trimester.

The optimal sensitive and specific cut-off value for FPG is still controversial [10]. The IADPSG and ADA have different views on the cut-off value at which a diagnosis is made. The IADPSG uses an FPG level of 5.10 mmol/l during the first prenatal visit and throughout pregnancy as the diagnostic criterion for GDM, whereas the ADA recommends that first trimester FPG should be used only to determine overt diabetes (7.00 mmol/L) and that the OGTT be used for GDM screening and diagnosis at 24–28 gestational weeks [5, 6]. A study of 6,520 pregnant women from India showed that a cut-off value of 76 mg/dL (4.2 mmol/L) for FPG had a highly sensitive and negative predictive value (NPV) for the diagnosis of GDM, whereas a cut-off value of 92 mg/dL (5.1 mmol/L) for FPG had a high specificity and positive predictive value (PPV) for diagnosis [14]. In a meta-analysis, 8 of 29 studies used different cut-off values for diagnosing GDM, and two reported 91 mg/dl (5.05 mmol/L) as the optimal cut-off value for diagnosis [10]. Other studies reported cut-off values of 81 mg/dl (4.5 mmol/L), 83 mg/dl (4.6 mmol/L), 84.5 mg/dl (4.69 mmol/L), 86.8 mmol/dl (4.82 mmol/L) and 89 mg/dl (4.94 mmol/L). In these studies, the sensitivity for most cut-off values was also in the range of 60–80% [9, 10, 18], similar to ours. Our study also suggests that first trimester FPG levels may be an indicator of subsequent GDM. Moreover, our research showed that the incidence of GDM increased with first trimester FPG levels. If FPG levels in pregnancy are not well controlled, the opportunity to reduce the risk of adverse outcomes is likely to be missed [11]. Impaired glucose tolerance usually occurs in the second trimester; only after this can treatment start. In fact, when these pregnant women were first assessed as having GDM, more than 20% of the foetuses showed signs of macrosomia, which may influence clinical decisions, such as the choice of delivery mode [10]. Screening for GDM in the first trimester can reduce the incidence of pregnancy complications, macrosomia, caesarean section and others [10, 14]. Therefore, early detection of GDM may reduce the risks and enable strict guidance from the beginning of pregnancy. Some studies found that despite treatment, patients diagnosed with GDM at an early stage had poorer outcomes, suggesting that first trimester FPG levels may be a marker of glucose tolerance before pregnancy and poor pregnancy outcomes [3].

In addition, lifestyle interventions to prevent GDM have been shown to be most effective in the first trimester [19]. In a meta-analysis of more than 11,000 pregnant women, Song et al. concluded that lifestyle interventions can prevent GDM only if implemented before the 15th week of gestation [20]. Research on late intervention has been generally disappointing, which means that it would be useful to have early pregnancy markers of GDM risk to determine who could benefit from early intervention [19]. Prevention studies have shown the positive prevention effects of diet on the incidence of GDM, LGA, SGA and preterm birth [21, 22]. The two-step diagnosis of GDM in the second and third trimesters provides a narrow intervention window [19]. When receiving treatment, 20% of the foetuses showed signs of macrosomia and increased abdominal circumference, again emphasizing the need for early risk markers. In this regard, first trimester FPG levels may be useful in selecting patients for early screening or LGA monitoring for GDM [19]. In addition, because metabolic changes during pregnancy result in a decrease in PG of approximately 2 mg/dL between six and ten gestational weeks, a specific threshold of PG for the gestational week or a narrow interval for the evaluation of first trimester FPG levels should be determined [23]. Therefore, we recommend that all pregnant women have FPG assessed at their first visit in the first trimester to determine the risk of diabetes and GDM.

## Conclusions

Based on our research, we recommend that all pregnant women undergo FPG testing in the first trimester, particularly at the first antenatal visit. Furthermore, we suggest that the risks of GDM should be given increased attention and management as soon as the first trimester FPG value is more than 4.7 mmol/L. The first trimester FPG should be considered a screening marker when diagnosing GDM in pregnant women, although it cannot replace the golden diagnostic standard of OGTT tests at present. A first trimester FPG level greater than 4.7 mmol/L should be considered a warning level. These factors may have a significant impact on the clinical treatment of pregnant women. These data come from southern China, and the results may apply only to pregnant women in southern China. Despite this, this study provided valuable insights into the accuracy of first trimester FPG levels in the screening and diagnosis of GDM in southern China.

### Limitations

Fagan’s nomogram showed that FPG is clinically useful [10]; the diagnostic effect of any screening test for GDM may depend on several other factors, such as ethnicity, the timing of testing and the presence or absence of risk factors for GDM [10]. In the absence of some missing data, we were unable to analyse the impact of some potential risk factors on the relationship between FPG and GDM. We did not have multiple data points from a single panel to indicate the most appropriate week for screening. So the conclusion that a first trimester FPG value ≥ 4.7 mmol/L is regarded as the management threshold of GDM needs to be confirmed by more prospective studies.

## Data Availability

All data generated or analysed during this study were included in this published article.

## Abbreviations

PG: Plasma Glucose
FPG: Fasting Plasma Glucose
GDM: Gestational Diabetes Mellitus
ACOG: The American College of Obstetricians and Gynecologists
WHO: World Health Organization
ADA: American Diabetes Association
IADPSG: International Association of Diabetes and Pregnancy Study Groups
OGTT: Oral Glucose Tolerance Test
